# Zinc treatment is efficient against *Escherichia coli* α-haemolysin-induced intestinal leakage in mice

**DOI:** 10.1038/srep45649

**Published:** 2017-03-31

**Authors:** Stephanie Wiegand, Silke S. Zakrzewski, Miriam Eichner, Emanuel Schulz, Dorothee Günzel, Robert Pieper, Rita Rosenthal, Christian Barmeyer, André Bleich, Ulrich Dobrindt, Jörg-Dieter Schulzke, Roland Bücker

**Affiliations:** 1 Institute of Clinical Physiology, Charité - Universitätsmedizin Berlin, Campus Benjamin Franklin, Berlin, Germany; 2 Institute of Veterinary Physiology, Freie Universität Berlin, Berlin, Germany; 3 Institute of Animal Nutrition, Department of Veterinary Medicine, Freie Universität Berlin, Berlin, Germany; 4 Institute for Laboratory Animal Science, Hannover Medical School, Hannover, Germany; 5 Institute of Hygiene, University of Münster, Münster, Germany

## Abstract

Zinc homoeostasis exerts protective effects in inflammatory intestinal diseases and zinc supplementation has been successfully used for treating infectious diarrhoea. This study aimed at a characterisation of zinc effects on focal leak induction by α-haemolysin (HlyA)-producing *Escherichia coli (E. coli)* as protective mechanism for colitis. We conducted *in vivo* experiments by oral challenge of gnotobiotic mice colonised with HlyA-expressing *E. coli*-536. Mice were either fed a defined normal or high zinc diet to analyse effects of zinc as a therapeutic regimen. HlyA-deficient *E. coli*-536 mutants were used as controls. Mice infected with HlyA-producing *E. coli* showed impaired barrier integrity when receiving normal zinc. High zinc supplementation in HlyA-producing *E. coli*-infected mice reduced epithelial dysfunction as indicated by ameliorated macromolecule permeability. Reduced size of focal leaks with diminished bacterial translocation was observed as inherent mechanisms of this zinc action. In human colon cell monolayers application of zinc rescued the HlyA-dependent decline in transepithelial electrical resistance via reduction of the calcium entry into HlyA-exposed cells. Calcium-dependent cell exfoliation was identified as mechanism for focal leak induction. In conclusion, zinc supplementation protects from HlyA-induced barrier dysfunction *in vivo* and *in vitro*, providing an explanation for the protective efficacy of zinc in intestinal disorders.

Zinc, an essential micronutrient, is involved in the regulation of multiple cellular functions and fullfills several key functions e.g. as a catalytic cofactor for enzymes or a structural cofactor for proteins. More than 300 enzymes and 1,000 transcription factors depend on zinc for their appropriate function. The innate immune system utilises zinc for antimicrobial actions (e.g. calprotectin). Zinc deficiency is therefore linked to the pathogenesis of several diseases^1^. Clinical studies have shown zinc deficiency in patients with liver diseases^2^, malabsorption syndrome^3^, and inflammatory bowel disease (IBD)^4^, whereas normal zinc levels largely contribute to intestinal function and health^1^. Zinc deficiency exacerbates experimental colitis in rats or mice (with 2 ppm zinc/day)^5^,^6^ and supplemental feeding with zinc ameliorates colitis and diarrhoea^5^,^6^. Additionally, dietary supplementation of 2,000 to 3,000 ppm of zinc oxide (ZnO) is commonly used as alternative to in-feed antibiotics, in order to alleviate *E. coli*-induced diarrhoea and improve growth performance in post-weaning piglets^7^,^8^,^9^.

The pathophysiology of intestinal disorders is closely linked to intestinal barrier function. One of the most important functions of the intestine is to provide a physical barrier against luminal noxious agents, such as bacteria, toxins and dietary antigens^10^,^11^. A continuous epithelial cell layer is essential to maintain epithelial barrier function. The tight junction seals the lateral intercellular space between adjacent epithelial cells against the lumen. Tight junction defects, an increase in epithelial apoptotic rate, the appearance of ‘focal leaks’ in the epithelium or of other gross lesions as ulcers, or a combination of these effects evoke intestinal barrier impairment. Such intestinal barrier defects increase the epithelial permeability to water and small solutes leading to ‘leak flux’ into the lumen (leak flux diarrhoea)^12^,^13^. Apart from that, a decreased restriction against macromolecules can lead to antigen influx into the mucosa, which provokes immune responses (leaky gut concept).

Dysbiosis, an abnormal microbiota composition, and decreased complexity of the gut microbial ecosystem are common features in patients with IBD^14^. Most strains of *Escherichia coli (E. coli*) are regarded as commensals in the gut. Distinct strains e.g. enteropathogenic *E. coli* (EPEC) or uropathogenic *E. coli* (UPEC) retain virulence factors such as fimbriae, enterotoxins, or serine protease autotransporters (SPATES, e.g. Sat in UPECs) which can cause intestinal and extra-intestinal dysfunctions^15^,^16^,^17^,^18^. *E. coli* strains of the B2 phylogroup are linked to urinary tract infection, but they frequently reside in the intestinal mucosa of patients with IBD^19^,^20^,^21^. One specific virulence factor of *E. coli*, the α-haemolysin (HlyA), appears to be a potentiator of inflammatory activity in the colon. *E. coli* HlyA pertains to the β-barrel-pore-forming toxin (β-PFT) group, it inserts itself receptor-independently into cell membranes^22^. It forms cation-permeable channels in the host cell membrane with an inner diameter of 20 Å^23^. Haemolytic bacteria have been shown to induce intestinal malabsorption^24^, secretion^25^ and epithelial lesions via necrosis and/or apoptosis induction^26^. We previously showed HlyA to induce ‘focal leaks’ in colonic HT-29/B6 cells. Within these epithelial lesions *E. coli* accumulated before translocation^27^. In mouse colon epithelium, HlyA-containing supernatants delayed epithelial restitution after induction of single-cell lesions, pointing to an inhibition of epithelial repair mechanisms^28^. Recently, we could show in mouse colonisation models that *E. coli* HlyA impairs intestinal barrier function *in vivo* via focal leak induction in the colon epithelium, thereby intensifying antigen uptake and triggering intestinal inflammation^29^. Our study aims to characterise the therapeutic potential of zinc in HlyA-induced gastrointestinal disorders with special attention on zinc’s ability to protect from intestinal focal leaks in the *E. coli* colonisation mouse model and in infected human epithelial cell lines.

## Results

### Zinc was protective against barrier defects from HlyA-producing *E. coli*-536

**Figure 1:**
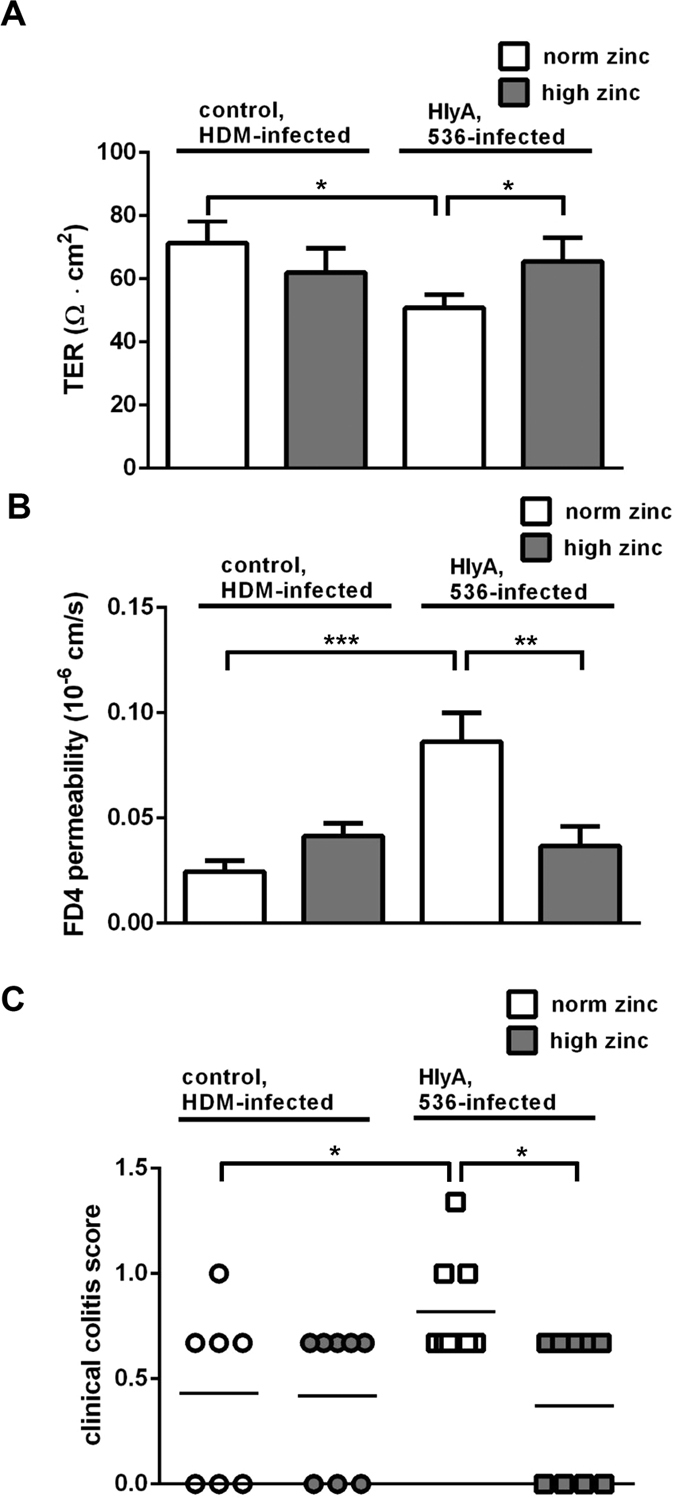
High zinc diet protects against HlyA-induced barrier defect in mouse colon. (**A**) Transepithelial electrical resistance (TER) was assessed to verify barrier integrity. The white bars display the overall transmural TER of the colon in ohm·cm^2^ of normal zinc-fed germ-free mice colonised either with the haemolysin-deficient *E. coli* mutant (HDM) as control or with the HlyA-producing *E. coli*-536. The dark bars represent the TER of the colon of high zinc-fed mice colonised in the same manner by the *E. coli* strains but receiving a therapeutically high zinc dose of 500 mg/kg. High zinc-treated groups displayed reduced epithelial defects indicated by increased TER, when compared to the respective physiological zinc-treated group. Both, zinc diet and bacterial colonisation took no longer than one week. Dynamite plots represent mean values and SEM over all mouse colon specimens. Each n = 5, *P < 0.05, Student’s *t*-test. (**B**) Zinc supplementation prevents an increase in epithelial permeability to 4 kDa FITC-Dextran (FD4) in HlyA-challenged mice. Mucosal to serosal permeability to macromolecule FD4 in mouse colonic epithelium after infection with HlyA^+^ *E. coli*-536 and simultaneous application of norm zinc (white bars) or high zinc diet (dark bars). *E. coli*-HDM served as control strain (n = 7). HlyA^+^ groups: n = 5, *P < 0.05, ***P < 0.001, Student’s *t*-test. (**C**) Clinical colitis score. Scoring depends on body weight, consistency of faeces and occurrence of blood in faeces. Bars represent mean values, dots and squares represent individual values; 0 (healthy) to 4 (maximal activity of colitis). HDM groups, n = 8 each; HlyA^+^ groups n = 9 each, *P < 0.05, Student’s *t*-test.

In Ussing chamber experiments, barrier integrity of the mouse colon epithelium was investigated. When a physiological zinc diet was given (norm zinc), a decrease in overall transepithelial electrical resistance (TER) was observed when animals were colonised with HlyA-harbouring *E. coli*-536 compared to mice colonised with the isogenic haemolysin-deficient mutant (HDM) strain (Fig. 1A). However, under a therapeutic zinc concentration (high zinc diet) which *per se* had no effect on TER, this decrease in TER after infection with HlyA-producing *E. coli* was abolished (Fig. 1A, *P* = 0.035). In addition to TER, macromolecule permeability measurements were performed with 4 kDa FITC-dextran (FD4) as an indicator of barrier defects such as gross lesions. In the physiological norm zinc group, FD4 fluxes were increased in *E. coli*-HlyA^+^-infected mice when compared to HDM controls (Fig. 1B, *P* = 0.0007). In mice receiving the high zinc dose which *per se* had no effect on FD4 flux, the increase in FD4 permeability in response to HlyA-producing *E. coli* was abrogated (Fig. 1B, *P* = 0.0171).

In order to characterize the inflammatory diarrhoea due to higher antigen influx, we assessed the clinical outcome of the colonisation with HlyA^+^ *E. coli* in our mouse model by means of a colitis score, depending on weight loss, consistency of the faeces and blood in faeces. An increase was seen in mice receiving the norm zinc diet and HlyA^+^ *E. coli* compared to mice with zinc supplementation (Fig. 1C, *P* = 0.0066).

### Assessment of luminal Zn^2+^ concentration

The amount of protein-bound and unbound zinc in the luminal content of the large bowel of the animals was determined by atomic absorption spectroscopy and evaluated in mg per kg of intestinal content. Mice receiving a norm zinc diet consisting of 15 mg zinc/kg chow gained an unbound portion of Zn^2+^ of 1.5 ± 0.3 mg/kg ( = 22 μM Zn^2+^) in the lumen and a bound amount of 30 ± 4 mg zinc/kg (n = 4). Mice fed a high zinc diet containing 500 mg zinc/kg chow had an unbound Zn^2+^ fraction of 29 ± 2 mg/kg ( = 460 μM Zn^2+^) and a protein-associated zinc of 831 ± 11 mg/kg intestinal content (n = 6). Thus, intestinal contents of mice fed high zinc diets contained a much higher reservoir of unbound Zn^2+^ ions (*P* < 0.0001) as potential inhibitor of the bacterial pore-forming toxin HlyA.

### No tight junction effects with zinc in HlyA^+^ *E. coli*-colonised mice

The overall mucosal integrity was not disturbed even after *E. coli* HlyA exposure, since there was no change in mucosal architecture. The H&E staining was without relevant pathology (data not shown). Furthermore, no alterations in tight junction protein expression were observed. Occludin expression in western blots was 108 ± 6% in the HlyA^+^-high zinc group versus 100 ± 36% in the HlyA^+^-norm zinc group (not significant, *P* = 0.85, n = 5 each). Barrier-forming tight junction proteins claudin-1 and claudin-3 were also unchanged between both HlyA^+^ groups (claudin-1: 89 ± 12% high zinc versus 100 ± 20% norm zinc, *P* = 0.64, and claudin-3: 116 ± 9% high zinc versus 100 ± 14% norm zinc, *P* = 0.38, n = 5 each).

### Zinc administration reduced focal leaks in α-haemolysin-producing *E. coli*-challenged mouse colon

**Figure 2:**
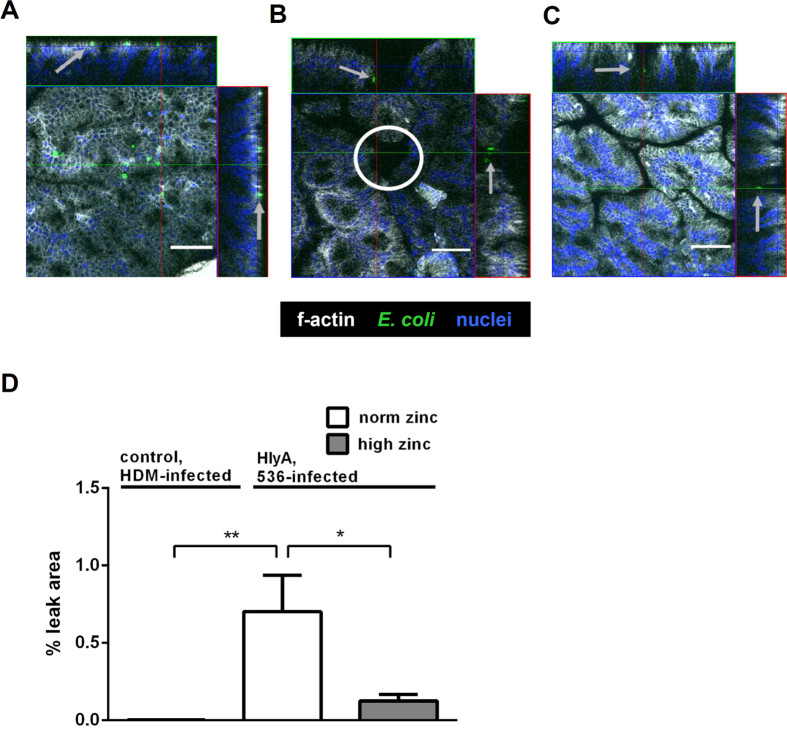
Zinc reduced number and size of *E. coli* HlyA-induced focal leaks in the mouse colon. Whole mount immunofluorescence staining of (**A**) normal colonic mucosa with apically attached HDM bacteria (arrows) without any cell damage as control condition, and staining of (**B**) physiologically zinc-fed mouse (norm zinc) colonic mucosa after *E. coli*-536-colonisation with one representative focal leak (white circle) and bacteria inside the lesion (arrows). A focal leak was characterised by shape estimation (funnel-shaped notch), co-localisation of *E. coli* signals (green) inside the lesion, and the absence of normal epithelial integrity (cytoskeletal F-actin = white, nuclei = blue). **(C)** In the high zinc group single invaded bacteria could be identified, but mostly leak induction was absent or small-sized. White bars = 50 μm. The lesions were digitally marked and measured with Zeiss LSM Image Examiner software for focal leak area size calculation as described previously^27^,^29^. (**D**) Quantification of leak area in % of the whole investigated area in a low-power field. The observation area of approximately 0.2 cm^2^ was screened microscopically for focal leaks. Identified leaks were digitally marked in a deep focus plane and the overall area of single leaks was divided by the overall observed specimen surface. In *E. coli*-HDM-treated mice focal leaks were not evident, whereas mice colonised with the HlyA^+^ *E. coli* displayed lesions. In the high zinc-supplemented group, the focal leak areas were reduced compared to the norm zinc group. n = 5, *P < 0.05, **P < 0.01, Student’s *t*-test.

We microscopically analysed un-sectioned mouse colon for lesions and bacterial signals using immunofluoresence (IF) whole mount stainings, in order to identify focal leaks as observed earlier^29^. The IF staining-based characterisation of lesions and/or erosions in the colonic mucosa of HlyA^+^ *E. coli*-colonised mice was assessed by shape estimation (funnel-shaped notch), co-localisation of invaded *E. coli* inside the lesion and the focal regions with disturbed epithelial surface integrity. Focal leaks could be detected in HlyA^+^ *E. coli*-colonised and norm zinc fed mice, but were absent in HDM-colonised mucosae. HlyA^+^ mice, receiving a high zinc supplementation, exerted a reduced focal leak size as well as a decreased overall number of focal leaks in comparison to the norm zinc group. For microscopic quantification, focal leaks were counted, digitally outlined, and sized. The quantification of leak size in mice infected with HlyA^+^ *E. coli* revealed a decrease after high zinc-feeding compared to the norm zinc-fed control group, whereas in HDM-colonised mice no leaks could be observed at all (Fig. 2). In the norm zinc-supplemented HlyA^+^ mice, one to three gross leaks with a size range between 1,600 and 15,000 μm^2^ and a mean leak area of 7,000 μm^2^ were measured per power field, which is 0.8% of the respective tissue area. No such leaks were detected in HDM-colonised mice. The high zinc mice showed a strong reduction in focal leak area to 0.1% when colonised with HlyA^+^ *E. coli* (Fig. 2D, HlyA^+^ versus HDM control: *P* = 0.0096, and HlyA^+^-norm zinc vs. HlyA^+^-high zinc: *P* = 0.0434).

### Zinc inhibited the translocation of intestinal *E. coli*

**Figure 3:**
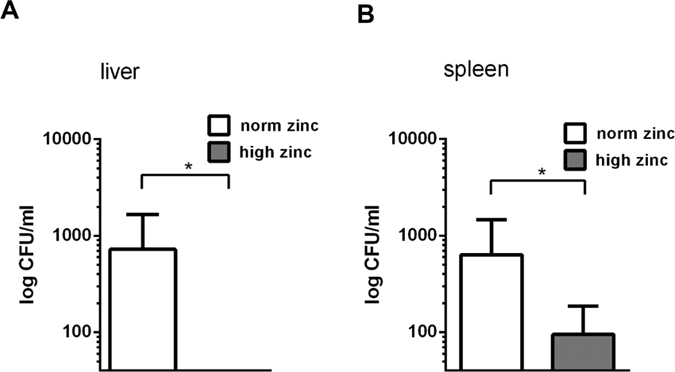
Zinc protects against translocation of intestinal *E. coli* to other organs. Bacterial translocation in mice was characterised by the plating method and colony counting. An enhanced bacterial invasion and translocation to **(A)** spleen and **(B)** liver was observed in *E. coli*-infected mice, which were norm zinc fed. Mice under norm zinc supplementation (white bar) were compared to the high zinc administered mice (dark bar). In order to avoid CFU counting mistakes as consequence of dilution, a threshold was put at 20 CFU as a lower detection limit. Each n = 5, *P < 0.05, Chi square test.

In a previous study, accumulation of B2 phylogroup *E. coli* was observed within focal leaks that had diameters from 5 to 50 μm and translocation of bacteria was detected through these focal leak areas^27^,^29^. In our present mouse model, liver and spleen were assessed for bacterial translocation by plating on blood agar plates and CFU (colony forming units) counting after 24 h. Diminished bacterial translocation in liver and spleen was observed in high zinc mice in comparison to the low zinc group (Fig. 3, both *P* < 0.0001).

The innate immune system includes cells and defence mechanisms that protect the host from infection by other organisms. During inflammation, calprotectin, an abundant neutrophil protein, defends other host cells with the function to release zinc and manganese as antimicrobial activity^30^. Employing the calprotectin assay, an initial inflammatory response in HlyA^+^ mice was measurable at day 2 to day 4 p.i., but did not reach significance versus the HDM control, since HDM mice also showed innate immune responses due to the *E. coli* colonisation (data not shown).

### Protective effect of zinc against HlyA-induced barrier damage in a human colon cell model

**Figure 4:**
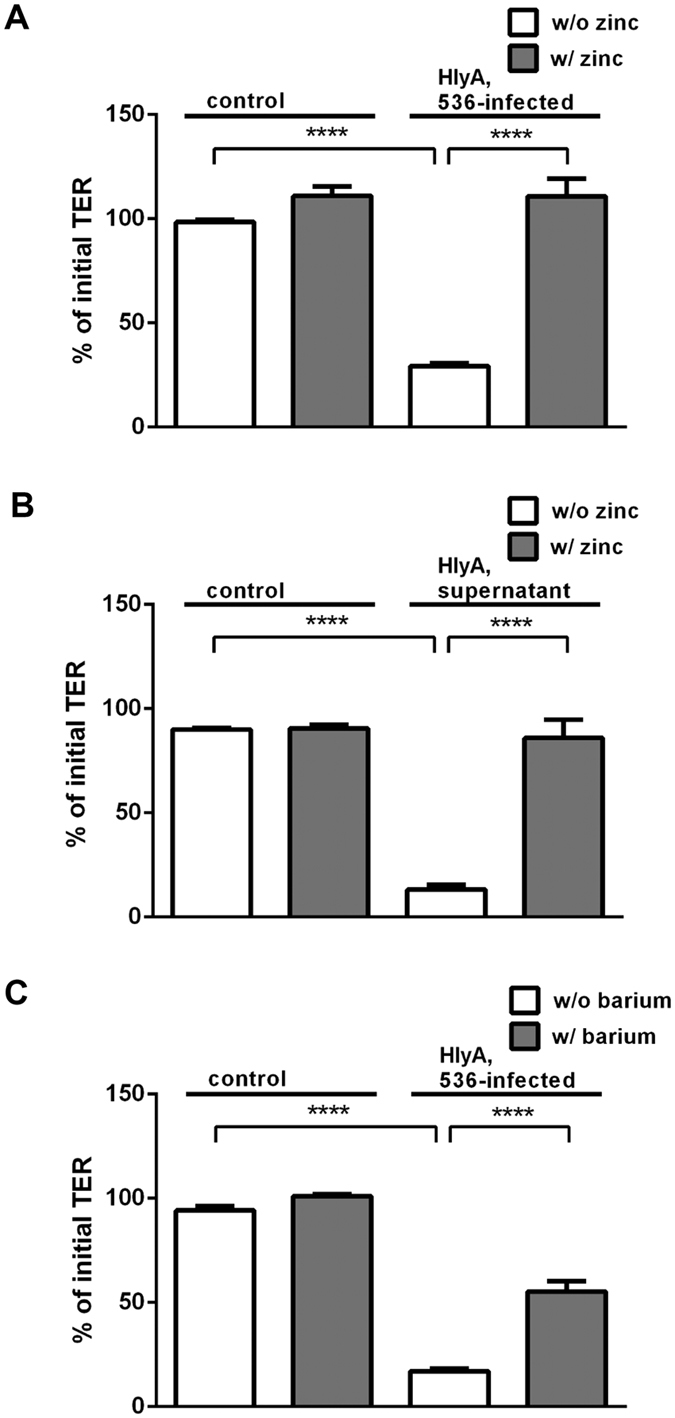
Protective effect of zinc and barium on HlyA-infected human colon cells. HT-29/B6 monolayers were apically inoculated for 4 hours with (**A,C**) 10^6^ CFU HlyA^+^ *E. coli*-536 bacteria per filter or with (**B**) 50 μl of concentrated HlyA-producing *E. coli*-536 supernatant and simultaneous addition of divalent cations compared to untreated monolayers with (w/) or without (w/o) (**A,B**) zinc or (**C**) barium addition. Each n = 6, ****P < 0.0005, Student’s *t*-test.

Without addition of zinc, barrier integrity analysis of HT-29/B6 monolayers *in vitro* revealed a decrease in TER when infected with HlyA-producing *E. coli*-536 compared to cells infected with the HDM control strain (*P* = 0.0001). When HT-29/B6 monolayers exposed to a dose of 500 μM zinc acetate were infected with HlyA^+^ *E. coli*, the TER decrease was inhibited compared to HlyA^+^ infected *E. coli* monolayers without zinc supplementation (Fig. 4A, *P* = 0.0001). In parallel experiments on the same HT-29/B6 cell model, an identical protocol was performed with concentrated supernatant from HlyA-containing *E. coli* instead of bacteria, simultaneously supplied with or without zinc. This resulted in identical effects on TER (*P* = 0.0001) and in identical inhibition by 500 μM zinc supplementation (Fig. 4B, *P* = 0.0002), which is a similar concentration of free zinc ions as achieved in the lumen of our mouse model in the high zinc group described above. Moreover, the simultaneous addition of another divalent cation i.e. barium (500 μM barium chloride) could rescue the TER drop in HlyA^+^ *E. coli* infected HT-29/B6 cells as well (Fig. 4C, *P* < 0.0001).

### Zinc inhibited the HlyA-induced increase in intracellular Ca^2+^ and the subsequent barrier defect

**Figure 5:**
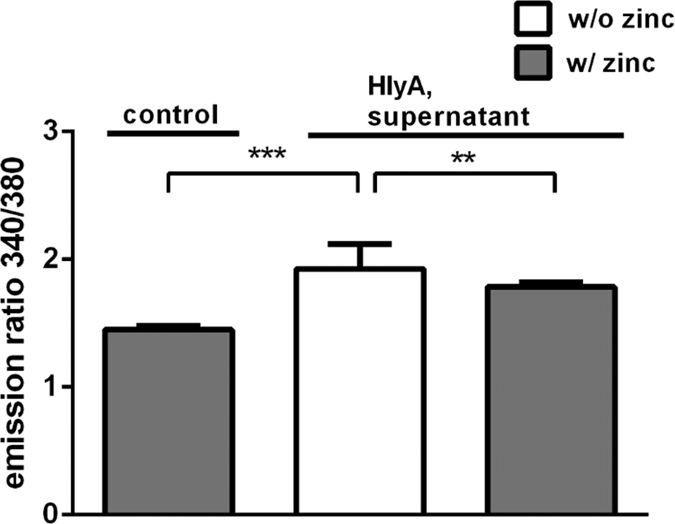
Zinc attenuates the HlyA-induced increase in intracellular Ca^2+^. Supernatant of HlyA-producing *E. coli*-536 was added apically to epithelial T84 cell monolayers and corresponding calcium levels were determined and compared to those of monolayers that were simultaneously incubated with supernatants of HlyA^+^ *E. coli*-536 plus Zn^2+^ (100 μM) compared to an incubation with supernatants of the HDM strain for 2 hours. The assessment for intracellular Ca^2+^ level was measured by Fura-2-AM and revealed a diminished Ca^2+^ influx when supplemented with Zn^2+^. n = 8, **P < 0.01, ***P < 0.001, Student’s *t*-test.

**Figure 6:**
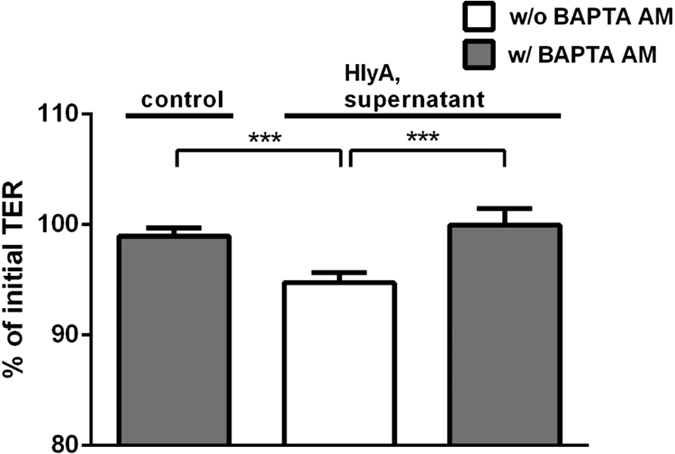
Prevention of barrier breakdown in HlyA-treated cells during inhibition with the Ca^2+^ chelator BAPTA-AM. HT-29/B6 monolayers were apically inoculated with 20 μl of concentrated HlyA-producing *E. coli*-536 supernatant with or without BAPTA-AM (10 μM) compared to uninfected BAPTA-AM monolayers. n = 3, ***P < 0.001, Student’s *t*-test.

In T84 colon cells, the increase in intracellular Ca^2+^ concentration ([Ca^2+^]_i_) after HlyA exposure (bacterial supernatant) was determined with or without addition of 100 μM zinc acetate using the fluorescent intracellular Ca^2+^ chelator Fura-2-AM. Without addition of zinc, treatment with HlyA led to an increase in [Ca^2+^]_i_ (*P* < 0.0001). This effect of HlyA was not seen anymore in the presence of zinc, where the Fura-2-AM signal was not increased and smaller than without Zn^2+^ (Fig. 5, *P* = 0.0082). In order to prove that the increment in [Ca^2+^]_i_ is as signalling event responsible for the pathogenic consequences towards a defective epithelial barrier, we tested the effect of inhibiting the HlyA-induced [Ca^2+^]_i_ increase on TER in our HT-29/B6 cell model with the intracellular Ca^2+^ chelator BAPTA-AM (Fig. 6). The initial decrease in TER after HlyA-exposure (*P* = 0.0033) could be entirely blocked by BAPTA-AM addition (*P* = 0.0068). Thus, the zinc-mediated inhibition of the increase in [Ca^2+^]_i_ could prevent the subsequent barrier breakdown.

### HlyA-induced epithelial cell shedding

**Figure 7:**
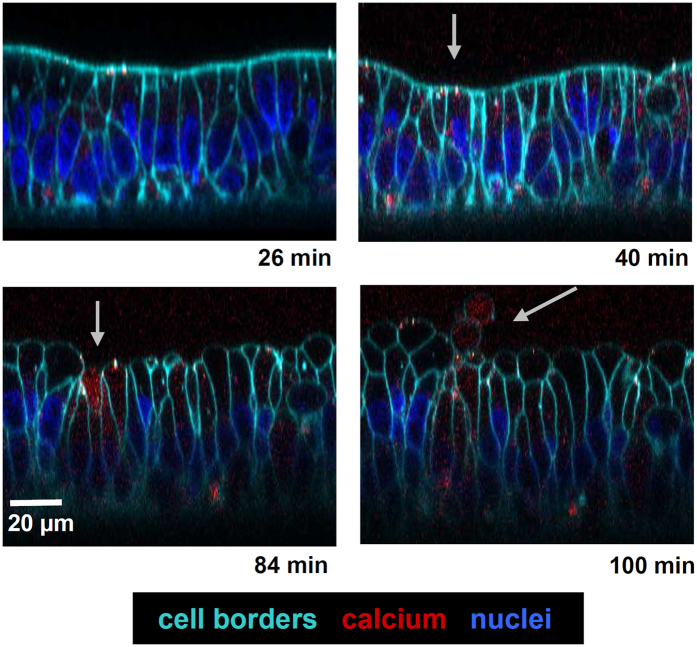
Cellular extrusion in HlyA-exposed HT-29/B6 monolayers after Ca^2+^ influx in live cell imaging. Lateral microscopic views on HlyA-incubated HT-29/B6 cell monolayer are given over time. Cell borders are stained turquoise with CellMask membrane stain and nuclei are stained blue with Hoechst staining. Cells were loaded with Fluo-4-AM dye which indicates calcium increase in the affected cells by red colour. Arrows indicate affected cells. Cells with red signals showed subsequently cellular extrusions leading to epithelial shedding (exfoliation). Bar 20 μm.

In live cell imaging of HlyA-exposed HT-29/B6 monolayers, cellular extrusions were observed after 100 min of incubation, leading to cell shedding off the epithelium (exfoliation). In the microscopic images of Fig. 7, an intracellularly increased calcium signal (red staining by Fluo-4-AM) was visualised prior to the extrusion. High calcium levels could be identified in HlyA-affected cells indicating calcium influx already at earlier time points (40 min in Fig. 7). This cell loss provides an explanation for the mechanism how focal leaks develop in the epithelium.

## Discussion

Impaired epithelial barrier function represents a principle feature in both, intestinal infections and inflammatory bowel disease such as in ulcerative colitis (UC). In UC, barrier dysfunction enables antigen entry into the mucosa, leading to inflammation in the leaky gut. Bacterial β-PFTs can play an important role in induction or maintenance of barrier defects in those diseases. It has been shown in a variety of studies that zinc supplementation ameliorates the duration and severity of diarrhoea and prevents subsequent episodes^31^,^32^. The World Health Organisation recommends to administer children 20 mg zinc for 10–14 days during diarrhoeal episodes^33^. The efficiency of zinc as a therapeutic regimen has been indicated in a DSS-colitis model in rats^5^. Furthermore, in patients suffering from Crohn’s disease a beneficial effect of zinc supplementation could be shown^34^, which is a key piece of evidence for the prevention of a *leaky gut* phenomenon by zinc.

Our study shows that zinc supplementation in a mouse model can inhibit HlyA^+^ *E. coli*-induced intestinal leakage. Additionally, we revealed the underlying mechanisms. Epithelial barrier integrity of mouse colon on a defined normal zinc diet (containing physiological zinc concentrations as explained under Methods) was impaired after HlyA^+^ *E. coli*-infection. However, when mice were fed a high zinc diet, barrier function could be preserved after HlyA^+^ *E. coli* colonisation. This also applies to the epithelial barrier function against larger molecules. Macromolecular markers like FD4, ovalbumin A (OVA) or horse radish peroxidase (HRP) are suitable to detect enhanced antigen influx through defective tricellular tight junctions or gross lesions like focal leaks or erosions^35^. In another animal model, colitis was induced by intra-rectal injection of acetic acid in piglets. In this model, the intestinal barrier integrity could also be rescued by diosmectite-zinc oxide supplementation as demonstrated by a decrease in FD4 fluxes^36^. The appearance of HlyA-induced focal leaks and the increase of the high-molecular antigen influx marker FD4 point to an onset of intestinal barrier dysfunction for antigens (“leaky gut”), which was represented by reduced colitis activity in our clinical colitis score in the HlyA^+^ group with zinc supplementation. This stresses our second main finding, namely zinc supplementation could also prevent the translocation of *E. coli* in mouse colon, a relevant mechanisms for the subsequent development of intestinal inflammation. Furthermore, mucosal or systemic clearance of bacteria can be enhanced by zinc supplementation as shown also by others, which could be due to different mechanisms including macrophage killing of bacteria under zinc supplementation^37^,^38^. As far as the signalling mechanisms for barrier preservation is concerned, zinc has been identified as an important factor for barrier integrity through regulation of occludin proteolysis and transcription of other tight junction proteins, i.e. claudin-3^39^. However, additional tight junction upregulation broadly across the epithelium could not be detected in our present experiments, but has been clearly shown in other models of zinc supplementation with different time frames^9^,^40^. Extracellular zinc and other divalent cations have been shown to protect erythrocytes from membrane damage from a variety of substances including β-PFTs^41^. Zinc ions have previously been shown to decrease the haemolytic actions of poly-alkylpyridinium polymer, a PFT from the marine sponge *Reniera sarai*^42^. A similar result was obtained in the present study. Zinc could attenuate the action of HlyA on transepithelial resistance as well as on Ca^2+^ entry. It is not clear so far, how zinc blocked the formation of pores or lesions by HlyA. Our *in vivo* findings point rather not to direct zinc effects on *E. coli* but to its secreted and externally activated HlyA. In principle, zinc effects could involve (i) influence on proliferation of *E. coli*, thus a bactericidal effect, or (ii) impact on the secretion process of the toxin and its oligomerisation leading to interferences in pore assembly and membrane integration, or (iii) inhibition of subsequent Ca^2+^-activated cytotoxic effects, triggered by HlyA^+^-induced Ca^2+^ influx or (iv) acceleration of lesion closure and restitution. All of the discussed mechanisms could cooperate for the protective mechanism of zinc on HlyA-induced lesions.

Concerning point (i), in our study zinc did not affect the proliferation or vital status of *E. coli* as proliferation assays revealed no bactericidal or bacteriostatic effect, as evaluated in pretests (see Methods section; bacterial infection model *in vitro*). An interaction between zinc and the bacterial cell membrane to prevent access of the toxin to the host membrane is conceivable. Gram-negative bacteria like *E. coli* possess an outer membrane composed of lipopolysaccharides (LPS), which is held together by magnesium and calcium ions that bridge negatively charged sugars. Addition of cationic peptides results in damage of the outer membrane and facilitates entry of molecules from the external environment (Shai-Matsuzaki-Huang model)^43^. Thus, zinc might prevent the capability of HlyA secretion via type 1 secretion system (T1SS). However, in our present study we can exclude influences on bacteria’s membrane or secretion system of the toxin referring to point (ii), because the TER effects of HlyA-containing bacterial supernatants on epithelial cells were still zinc-sensitive. Thus, zinc may interact with HlyA directly to interfere with its biological activity at the host cell membrane. Previous work on erythrocytes has suggested that Zn^2+^ and other divalent cations (Hg^2+^) as well as trivalent cations (La^3+^) can close pores formed by PFTs but cannot inhibit lysis caused by hypo-osmotic shock^41^. The cytotoxic effect of HlyA is massively amplified by ATP release presumably through the HlyA pore^44^ with subsequent P2X receptor activation. In erythrocytes, P2X1 and P2X7 receptors have been implicated in HlyA-induced haemolysis and blocking either of these receptors substantially reduced haemolysis^45^,^46^. Interestingly, insertion of a HlyA pore does not cause immediate cell swelling and rupture, but HlyA triggers acute erythrocyte shrinkage which depends on Ca^2+^-activated efflux of K^+^ via KCa3.1 and of Cl^−^ via TMEM16A^47^. In our cell model, Fura-2-AM was used to measure changes in intracellular [Ca^2+^] after HlyA-induced pore formation. When zinc was added, Ca^2+^ influx was reduced. Combined with the electrophysiological data, the studies with Fura-2-AM indicate that zinc reduced focal leak area by inhibiting Ca^2+^ entry, strongly supporting the mechanism of point (iii). And indeed, calcium signalling led to epithelial cell loss (exfoliation) by induction of cell death which in our cell model was directly shown by detection of cellular extrusions in live cell imaging. Cells with higher [Ca^2+^]_i_ levels ended up in cellular shedding. This calcium-dependent exfoliation might finally lead to focal leaks as a mechanistic explanation, which is the third important finding of this study. Thus, as shown by others before^48^ high [Ca^2+^]_i_ has to be assumed to lead to cell death (via necrosis, autophagy or anoikis) also in our study. Rapid calcium oscillations have earlier been described in renal epithelial cells in response to UPEC HlyA that could be linked to a pathological mechanism in pyelonephritis^49^. Moreover, extensive shedding of the uroepithelium and haemorrhage in urinary bladder tissue in mice have been observed during HlyA-positive UPEC infections. Also those morphological changes in the epithelium might be linked to Ca^2+^ dysregulation^50^. On the other hand, the colon mucosa of HlyA^+^ *E. coli*-colonised mice showed no changes in epithelial architecture and also no change in tight junctions broadly across the epithelium or apoptotic cell death rate was observed after B2 phylogroup *E. coli* colonisation neither in our present nor in our earlier observations^27^,^29^. That focal leak disturbance is sufficient to explain the TER effect in our mouse model was already calculated in one of our previous manuscripts (see supplemental calculation in ref. 29). Thus, taken together the effects measured here rather reflect the focal features of the *E. coli* cytotoxicity.

Similar features regarding reduced epithelial resistance and increased active anion secretion have been shown for the haemolysin aerolysin (AerA) from *Aeromonas hydrophila*, although with slower kinetics^51^. Moreover, in AerA-treated HT-29/B6 monolayers an intracellular calcium increase was found to activate myosin light-chain kinase. This in turn results in a constriction of the perijunctional cytoskeleton with subsequent tight junction protein redistribution and inhibition of cellular restitution from epithelial lesions.

That leads us to point (iv) of the mechanisms of the putative zinc actions which is supported by two independent pieces of evidence. First, restitution from experimentally induced small epithelial lesions was retarded by *E. coli* HlyA in mouse colon^28^. Secondly, zinc could indeed restore the defective recovery from epithelial single cell lesions in HT-29/B6 cell monolayers after AerA exposure^52^, which is a direct hint for the existence of this type of protective cellular mechanism.

Other infectious bacteria e.g. *Staphylococcus aureus* secrete α-haemolysins with high structural similarities to *E. coli* α-haemolysin. In urinary bladder epithelial cells these α-haemolysins were shown to induce potassium efflux but also calcium influx with subsequent inactivation of Akt/protein-kinase B signalling, mediating cell survival^53^. In our HT-29/B6 cell model infected with *E. coli* HlyA, we rather observed Akt/protein kinase B phosphorylation (activation) by HlyA (supplement in ref. 29). Studies on pores formed by a variety of amyloid peptides from neurodegenerative disease and other malignancies have demonstrated a blockade of pores by zinc with a lack of ion selectivity and voltage dependency. The molecular structure of amyloid pores resembles the β-barrel structure of PFT’s formed by bacterial toxins such as staphylococcal α-haemolysin, anthrax toxin, and clostridial perfringolysin^54^. The food-poisoning bacterium *Clostridium perfringens* type A produces an enterotoxin (CPE) that induces diarrhoea. CPE is able to form cation-permeable pores in the apical membrane of human intestinal epithelial CaCo-2 cells. CPE mediated short-circuit current could be inhibited by zinc ions^55^. Thus, our study confirms previous studies on bacterial pathology from PFTs, the damaging effects of which were effectively inhibited by adequate inhibitory concentrations of the micronutrient zinc. The mechanism behind the inhibitory effect of zinc on HlyA from *E. coli* was already proposed in earlier studies as a mutually exclusive competition between zinc and calcium for binding sites on the HlyA leading to specific conformation changes with an inhibition of pore formation by zinc^56^,^57^. As a limitation of our present study calcium measurements were not possible in the mouse intestine *in vivo*. Therefore, we adapted the cell T84 model for these measurements. Finally, molecular resolution of the competitive binding of zinc and calcium to the HlyA would need greater effort as e.g. site-directed mutagenesis of HlyA calcium-binding sites which is beyond the scope of the present paper.

In conclusion, our study reveals that zinc supplementation can attenuate HlyA^+^ *E. coli*-induced barrier defects in the colon and thereby decreases bacterial translocation and antigen entry through the barrier disturbed colonic epithelium, attenuating immune response induction (leaky gut concept). These findings indicate that therapeutic zinc supplementation could be suitable to improve barrier defects induced by PFTs, thereby preventing enteric complications from infections such as post-infectious irritable bowel syndrome or chronic inflammation with extra-intestinal complications as reactive arthritis.

## Methods

### Bacterial culture and intestinal colonisation model

HlyA-producing *E. coli*-536 and *E. coli*-536-HDM (*E. coli*-536ΔhlyI_ΔhlyII: cat)^58^ were cultured in Luria-Bertani (LB) broth at 37 °C overnight (o/n) and grown to log-phase. Eight to twelve weeks old germ-free C3H/HeJ wild type mice were originally purchased from Jackson Laboratories (Bar Harbor, ME., USA). The mice were bred and housed as germ-free animals at the Hannover Medical School (Laboratory Animal Science). For colonisation experiments, the animals were housed under cleanroom conditions, given free access to sterile water and sterile mouse chow with either defined normal zinc dosage from zinc hydroxide carbonate ([ZnCO_3_]_2_·[Zn(OH)_2_]_3_) with 15 mg zinc/kg chow or high zinc dosage with 500 mg zinc/kg chow (ssniff Spezialdiäten GmbH, Soest, Germany). For intestinal colonisation, *E. coli*-536 or *E. coli*-536-HDM o/n cultures were diluted in LB broth to 10^5^ CFU/100 μl and administered via oral gavage (200 μl) to the mice^29^. The mice were maintained on normal or high zinc for 2 days before colonisation and throughout the incubation period of another 2 to 5 days. Standard laboratory mouse food pellets contain zinc supplementation of around 50 mg zinc/kg feed. Our defined normal zinc chow with 15 mg zinc/kg contains less but still a physiological zinc concentration, since in most diet components whether for mice or humans the zinc level ranges from 2 mg/kg (fruits, vegetables) up to 40 mg/kg (nuts, cereals). An experimental zinc deficiency, however, was excluded with 15 mg zinc/kg in our mouse model, since in mice zinc deficiency is only achieved in long-term protocols with application of chows under 10 mg zinc/kg. For example, Beach *et al*. used zinc diets for mice with 9 mg/kg for induction of marginal zinc deficiency, with 5 mg/kg for moderate zinc deficiency and 2.5 mg/kg for severe zinc deficiency^59^,^60^ and Sturniolo and coworkers induced experimental zinc deficiency by 2–3 mg/kg ( = 2–3 ppm) zinc per day^5^,^6^.

Clinical colitis score was assessed as described previously^61^. Briefly, no weight loss scored as 0, weight loss of 1–5% from baseline as 1; 5–10% as 2; 10–15% as 3; and more than 15% as 4. For stool consistency, a score of 0 points was assigned for well-formed pellets, 2 points for pasty and semi-formed stool pellets that did not adhere to the anus, and 4 points for liquid stools that did adhere to the anus. For bleeding, a score of 0 points was assigned for no blood, 2 points for positive haemoccult, and 4 points for gross bleeding. These scores were added together and divided by three, resulting in a total score of 0–4. The study was carried out in accordance with the German Animal Protection Act and the ARRIVE guidelines. The study was approved by the ethics committee for animal welfare in Berlin, Landesamt für Gesundheit und Soziales (LAGeSo Berlin) under the LAGeSo approval number G0273/14. Mice were sacrificed within one week after infection. After body weight monitoring of each mouse, the intestine was removed and directly used for electrophysiological measurements in Ussing chambers. In parallel, further samples were fixed in paraformaldehyde for histological staining. Specimens of faeces, spleen, and liver were plated in different dilutions on blood agar plates for CFU counting. Intestinal content from the large intestine (caecum, colon) was removed to determine the luminal bound and unbound zinc concentrations.

### Analysis of Zn^2+^ concentrations in the intestinal content

Total zinc of the caecal content was determined after hydrolysis of sample fractions in hydrochloric acid (37%) for 90 min at 250 °C in an atomic absorption spectrometer (AAS vario 6, Analytik Jena, Germany). To determine total insoluble zinc, samples were homogenised at room temperature (RT) for 1 h, diluted (1:2) in double-distilled water and centrifuged at 14,000 × g for 10 min. Supernatants were withdrawn, added on polymeric reversed-phase sorbent columns (8B-S100-FBJ, Phenomenex, Aschaffenburg, Germany) and the eluents were examined for the total free inorganic zinc amount. After elution with acetonitrile/water (4:6), acetonitrile/formic acid (7:3), and evaporation of the organic phase by vacuum centrifugation, protein-associated zinc was defined.

### Transepithelial electrical resistance and macromolecule marker fluxes

Colon samples from mice were mounted in Ussing chambers in physiological Ringer-type solution with substrate (beta-hydroxybutyric acid, l-glutamine, D(+)-mannose, and D(+)-glucose). Measurement of total transmural resistance was recorded under voltage clamp conditions by a computerised automatic clamp device (Fiebig-Hard&Software, Berlin, Germany). Subtracting resistance of the bathing solution between the voltage-sensing electrodes provided the corrected resistance values. Unidirectional flux measurements were conducted from mucosal to serosal direction. Fluorescein isothiocyanate-labelled (FITC)-dextran 4000 Da (FD4, Sigma-Aldrich, St. Louis, MO., USA) was added to the mucosal side of the tissue achieving a final concentration of 0.2 mM. To the serosal side unlabelled dextran 4000 Da solution was added at the same concentration. Samples were taken from the basolateral hemichamber in 30 min intervals and fluorescence was measured in a spectrofluorimeter (Tecan, Maennedorf, Switzerland). Permeability was calculated from flux over concentration difference.

### Visualisation of *E. coli* HlyA and epithelial leak areas by immunofluorescence microscopy

After dissection, colon samples were submerged in 2% paraformaldehyde for 3 h without rinsing the luminal content to conserve the localisation of the bacteria. Adding 25 mM glycine in PBS (phosphate buffered saline) quenches the protein linking. To assess the entire colon epithelium, whole mount staining was performed as described previously^27^. Colon tissues were permeabilised with 1% TritonX-100 in PBS for 2 h at 37 °C and subsequently incubated at RT for 3 h in blocking solution (10% goat serum, 1% bovine serum albumin, 0.8% TritonX-100 in PBS). Incubation with the primary antibody in blocking solution rabbit-anti-*E. coli*-O (1:200, provided by Lothar Beutin^27^,^29^) was performed o/n at 4 °C. The tissues were washed four times with blocking solution with a prolonged washing time for 1 h each, then incubated o/n at 4 °C with a secondary antibody (Alexa Fluor goat anti-rabbit IgG, 1:500; Invitrogen, Karlsruhe, Germany), followed by additional washing steps (four times with PBS for 30 min). Staining of nuclei with 4′,6-diamidino-2-phenylindole (DAPI) and cytoskeletal F-actin with Phalloidin Alexa Fluor647N was performed at RT for 30 min (each 1:1000). Stained tissues were mounted using ProTaqs MountFlour (Biocyc, Luckenwalde, Germany) and visualised by confocal laser-scanning microscopy (Zeiss LSM510, Jena, Germany) with a 40× water-immersion objective (2 mm working distance for deep optical *XY*-plain sections in *Z*-stacks). Western blotting and densitometry for quantification of tight junction protein expression were performed as described previously^29^.

### Epithelial cell culture and *in vitro* bacterial infection model

Human HT-29/B6 colon carcinoma cells^62^ were cultured in 25 cm^2^ culture flasks (Nunc, Denmark) in culture medium (RPMI 1640; Biochrom KG, Berlin, Germany; Gibco, Paisley, UK) supplemented with 1% penicillin/streptomycin and 10% fetal calf serum. T84 (ATCC CCL-248) colonic epithelial cells were cultured in a 1:1 mixture of Dulbecco’s modified Eagle’s medium and Ham’s F-12 medium supplemented with 10% fetal calf serum, 1% penicillin-streptomycin (Sigma-Aldrich, Germany). Culture was performed at 37 °C in a 95% air and 5% CO_2_ atmosphere. Cells were seeded on Millicell PCF filters (Millipore, MA, USA; membrane area 0.6 cm^2^; pore size 0.4 or 3 μm) at an average concentration of 7 × 10^5^ cells/cm^2^ and were grown until forming a confluent monolayer. *E. coli*-536 and *E. coli*-536-HDM log-phase cultures were re-suspended in LB broth. To exclude any prior Zn^2+^-dependent bacteriostatic or bactericidal effects in the culture, we performed proliferation assays proving the lack of any effects on bacterial growth (initial *E. coli* 536 concentration of 8 × 10^5^ per ml LB broth grew up to 4 × 10^7^ per ml LB broth in 6 hours, n = 3, *P* > 0.05). For infection of human epithelial cell monolayers the bacteria were diluted to 10^6^ CFU in LB broth, centrifuged and re-suspended in respective epithelial cell culture medium. For inhibition experiments, zinc acetate dihydrate (500 μM) was added simultaneously with the bacterial infection. For preparation of the concentrated supernatant containing HlyA from *E. coli-*536, the bacteria were cultured on blood agar plates at 37 °C o/n, afterwards re-suspended in cell culture medium and grown to log phase. The bacterial supernatant was collected by centrifugation (3,000 × g for 5 min) and concentrated via ultrafiltration (Amicon 50 k, Ultra Centrifugal Filter Units, Merck Millipore, Billerica, MA., USA). Zinc and HlyA was applied simultaneously to the cell monolayers.

### Transepithelial resistance of epithelial colon cell lines

Total transepithelial resistance of the colonic epithelial monolayers grown on permeable supports were measured as described^63^. Electrical measurements were performed in the culture dishes by a fixed pair of electrodes and an Ohmmeter (EVOM, World Precision Instruments, Sarasota, FL., USA). TER was measured from the voltage deflections caused by an external rectangular current pulse (±10 μA, 21 Hz). The temperature was maintained at 37 °C during the measurements. Resistance values were corrected for the resistance of the filter and the bathing medium^63^.

### Assessment of intracellular calcium levels in human epithelial cells

Changes in intracellular Ca^2+^ concentration after adding HlyA and zinc acetate to colon cell lines were assessed with ratiometric calcium measurement using the fluorescent dye Fura-2-AM (Invitrogen, Life Technologies). The ratio of fluorescence intensity emitted at 510 nm after excitation at 340 nm to the one emitted at 510 nm after excitation at 380 nm (340/380) reflects changes in intracellular Ca^2+^ concentrations. T84 cells were grown to confluency in Poly-L-lysin-coated 96 well plates, washed with HEPES/Krebs solution containing 2 g/l glucose and 2 mM probenecid (HEPES = 4-(2-hydroxyethyl)-1-piperazineethanesulfonic acid). Probenecid is an inhibitor of organic anion transporters located in cell membranes. These transporters often extrude fluorescent indicators from cells, and thereby contribute to poor dye retention. This phenomenon usually causes high background in the assays that require good retention of dye indicators inside cells. The use of probenecid to inhibit the activities of transporters and thus to reduce leakage of intracellular dye indicators is a common method for reducing fluorescence background of calcium assays. After 1 h of incubation with Fura-2-AM at 37 °C (25 μM, 2 mM probenecid, in HEPES with 2 g/l glucose), remaining extracellular Fura2-AM was washed away thoroughly (2 × 2 min and 2 × 5 min at 37 °C). Measurements were performed in 100 μl volume at each well in a plate reader (Tecan infinite 200, Research Triangle Park, NC., USA) at 37 °C. After baseline assessment, fresh buffer containing haemolysin or/and 100 μM zinc acetate (dihydrate) was/were added in replacement of previous used buffer solution. HDM supernatant-treated cells served as controls. Zinc and HlyA was applied simultaneously. Fura-2-AM has been proved to be useful in measuring small increases in intracellular zinc^64^ because of its high affinity for zinc, which is about 100 times greater than that for Ca^2+^ 65. In order to rule out competitive zinc interactions with Fura-2-AM with the consequence of a reduced Ca^2+^ detection, we conducted Ca^2+^ measurements with the calcium-specific Fluo-Zin3 that revealed no interference between Ca^2+^ and Zn^2+^ detection in Fura-2-AM measurements (data not shown).

### Live cell imaging

HT-29/B6 cells were seeded to the bottom of inverted filter supports (Millicell 12 mm; 0.4 μm pore size; Millipore; Germany) and imaging was performed on day 7 after confluency. Cells were washed once with HEPES/Ringer buffer (containing 2 g/l glucose, 2 mM probenecid, 1% penicillin/streptomycin). Subsequently, cells were loaded with the calcium dye Fluo-4-AM (4 mM Fluo-4-acetoxymethyl ester, Invitrogen) and the nucleus stain Hoechst 33342 (2 μM, Thermo Fisher Scientific, Waltham, MA., USA) for 1 h at 37 °C. For visualisation of cell borders 5 μg/ml CellMask plasma membrane stain (Invitrogen) was added. After washing thoroughly (3 × 5 min) with buffer, a first image was taken by confocal laser-scanning microscopy using a 40x water immersion objective (Zeiss LSM780, Jena, Germany) and maintained for 26 min with HlyA. After this first incubation period, images were taken every 2 min throughout the experiment with HlyA. The incubation chamber was heated to 37° and maintained with 5% CO_2_ in ambient air throughout the experiment.

### Statistical analysis

Data are expressed as mean values ± standard error of the mean (SEM). Statistical analyses were performed using the 2-tailed Student’s t-test or the Chi-square test as indicated. For multiple comparisons *P* values were Holm-Bonferroni adjusted. *P* < 0.05 was considered statistically significant.

## Additional Information

**How to cite this article**: Wiegand, S. *et al*. Zinc treatment is efficient against *Escherichia coli* α-haemolysin-induced intestinal leakage in mice. *Sci. Rep.* **7**, 45649; doi: 10.1038/srep45649 (2017).

**Publisher's note:** Springer Nature remains neutral with regard to jurisdictional claims in published maps and institutional affiliations.
